# Comparison of tibiofibular syndesmosis stability following treatment of proximal, middle, and distal third fibula fractures

**DOI:** 10.1007/s00590-024-04148-6

**Published:** 2025-01-11

**Authors:** Sean Thomas, Garrett Berger, Brendan O’Leary, Zachary Brumm, Alexandra K. Schwartz, William T. Kent

**Affiliations:** 1 School of Medicine, University of California San Diego, La Jolla, CA USA; 2 Department of Orthopaedic Surgery, University of California San Diego, 200 West Arbor Drive MC 8894, San Diego, CA 92103 USA

**Keywords:** Maisonneuve fracture, Tibiofibular syndesmosis, Syndesmotic-only fixation, Syndesmosis injury

## Abstract

**Purpose:**

While treatment modalities for Maisonneuve fractures involving the proximal third of the fibula are established, no studies to date have reported outcomes associated with syndesmotic-only fixation of middle third fibular shaft fractures. The purpose of this study was to evaluate outcomes associated with syndesmotic-only fixation in the treatment of Maisonneuve fractures involving the middle third of the fibula.

**Methods:**

A retrospective review was conducted on 257 cases of syndesmotic ankle instability with associated fibular fractures at a level 1 trauma center between 2013 and 2023. Patients were divided into cohorts based on fibular fracture location in the proximal, middle, or distal third of the fibula. The Chi-square test of independence, two-sample t-test, and analysis of variance were used to compare outcome measures between cohorts.

**Results:**

Sixty-six patients were identified including 48% (*n* = 32) with proximal third fibular fractures, 20% (*n* = 13) with middle third fibular fractures, and 32% (*n* = 21) with distal third fibular fractures. Rates of infection, loss of reduction, wound healing complications, and reoperation did not vary significantly between cohorts. Functional outcome measures including range of motion, time to weight-bearing, and tibiofibular/medial clear space measurements at final follow-up were similar across cohorts.

**Conclusion:**

Patients with Maisonneuve fractures involving the middle third of the fibula demonstrated positive outcomes with syndesmotic fixation alone, with no documented cases of infection, loss of reduction, or wound healing issues. By demonstrating maintenance of anatomic reduction and low rates of complications, our results support the use of syndesmotic-only fixation in the treatment of middle third Maisonneuve fractures.

## Introduction

The distal tibiofibular syndesmosis is an essential stabilizer of the ankle joint and is composed of three primary ligamentous structures: the anterior inferior tibiofibular ligament, the interosseous ligament, and the posterior inferior tibiofibular ligament [1]. Injuries to the ankle syndesmosis are common, occurring in 1%—18% of ankle sprains and 13%—50% of ankle fractures [2]. Instability of the syndesmosis is an indication for surgical stabilization, which can be achieved with screw fixation or a suture button device [3].

Syndesmosis injuries typically result from excessive external rotation or a combination of ankle dorsiflexion with adduction or abduction of the foot [4]. Lateral rotation of the talus within the mortise leads to sequential disruption of one or more syndesmotic ligaments. These events are often accompanied by a syndesmotic or supra-syndesmotic level fracture of the distal fibula [5]. In this scenario, internal fixation of the fibula alongside syndesmotic fixation is generally recommended to restore anatomic alignment of the mortise [6].

Maisonneuve fracture complexes are a rare injury pattern characterized by a proximal fibular fracture, distal tibiofibular syndesmosis disruption, and a medial malleolar fracture or deltoid ligament rupture [7]. In these injuries, external rotation of the talus strains the medial column of the mortise, leading to rupture of the anterior tibiofibular and interosseus ligaments. Transmission of this force anteriorly through the interosseous membrane results in a proximal fibula fracture [8].

While lateral plating of distal fibular fractures is often required to restore fibular length and syndesmosis alignment, Maisonneuve fractures involving the proximal third of the fibula are generally treated with syndesmotic-only fixation [7]. Anatomic alignment of the mortise can often be achieved through closed reduction of the fibula, and lateral plating proximally would require dissection of the peroneal nerve [9]. For these reasons, syndesmotic-only fixation has become the standard in proximal Maisonneuve fractures.

While treatment modalities for proximal Maisonneuve and distal third fibular fractures are established, no studies to date have reported outcomes associated with syndesmotic-only fixation in Maisonneuve fractures involving the middle third of the fibula. Lateral plating in these injuries requires a larger skin incision due to the deeper approach required to reach the fibula. The ability to achieve adequate reduction of the syndesmosis without fibular fixation has not been determined. The purpose of this study was to evaluate outcomes associated with syndesmotic-only fixation in the treatment of Maisonneuve fractures involving the middle third of the fibula.

## Materials and methods

After obtaining institutional review board (IRB) approval, a retrospective chart review was conducted at a level 1 trauma center on operative unstable ankle fractures from November 2013 to March 2023. Exclusion criteria included patients with Danis-Weber type A and B injuries, patients who did not undergo screw fixation for syndesmotic instability, and patients with less than 3 months of follow-up [10].

Patients were divided into three cohorts based on fibular fracture location in the proximal, middle, or distal third of the fibula. Fibular fracture location, treatment, radiographic data, postoperative complications (including infection, loss of reduction, wound healing issues, and reoperation), follow-up duration, and range of motion measurements at final follow-up were recorded. To analyze maintenance of syndesmotic reduction postoperatively, anteroposterior and oblique ankle radiographs were measured to determine medial clear space (MCS) and tibiofibular clear space (TFCS) values. Changes in these parameters were evaluated at first and final follow-up appointments.

The Chi-square test of independence was used to compare postoperative complications across groups. MCS and TFCS values were compared using a two-sample t-test and analysis of variance (ANOVA). Plantar flexion and dorsiflexion range of motion (ROM) measurements were recorded at final follow-up and compared between groups using ANOVA. All statistical tests were analyzed with significance set at *p* < 0.05.

## Results

There were 257 cases of operative ankle injuries with associated fibular fractures identified. Of these, 43 were excluded for Danis-Weber type A or B classifications, 21 for not undergoing screw fixation for syndesmotic instability, and 127 for having less than 3 months of follow-up, summing to 66 patients. Forty-eight percent (n = 32) of these had proximal third fibular fractures, 20% (n = 13) had middle third fibular fractures, and 32% (n = 21) had distal third fibular fractures. The average follow-up duration across cohorts was 7 months. The proximal fibula cohort had an average follow-up length of 6 months, the middle fibula cohort 5 months, and the distal fibula cohort 11 months (Table 1).

**Table 1:** Clinical data and outcomes

Clinical data	Proximal fibula	Middle fibula	Distal fibula
Total patients	32	13	21
Average follow-up	6 months	5 months	11 months
Mean time to WBAT	2.5 months	2.5 months	2.7 months
Delayed wound healing	0 (0%)	0 (0%)	0 (0%)
Infection	0 (0%)	0 (0%)	0 (0%)
Loss of reduction	0 (0%)	0 (0%)	0 (0%)
Reoperation	5 (16%)	1 (8%)	3 (14%)

WBAT = weight-bearing as tolerated

All injuries demonstrated syndesmotic instability which was addressed through screw fixation. In the proximal and middle third cohorts, screw fixation occurred with two 3.5-mm cortical screws with three or four cortices of fixation. Screw tightening was performed with the ankle in dorsiflexion, and the most distal screw was placed a mean 2.10 cm proximal to the joint line. Injuries to the deltoid ligament were treated non-operatively. All proximal and middle third fibular fractures were treated closed, and all distal third fibular fractures underwent plate fixation. Reoperation for symptomatic hardware removal was observed in 16% (*n* = 5) of proximal fibular fractures, 8% (*n* = 1) of middle third fibular fractures, and 14% (*n* = 3) of distal third fibular fractures. There were no instances of infection, loss of reduction, or wound healing issues observed (Table 1). Rates of reoperation did not vary significantly between cohorts.

Ankle plantar flexion at final follow-up was greater in the distal third fibula cohort (34°) when compared to the proximal and middle third cohorts (28° and 29°, respectively) (*p* = 0.03). There was no significant difference in ankle dorsiflexion between cohorts. Average widening of the TFCS across follow-up was measured at 0.50 mm (*p* = 0.02) in the proximal fibula cohort, 0.02 mm (*p* = 0.48) in the middle fibula cohort, and 0.67 mm (*p* = 0.003) in the distal fibula cohort. Average MCS widening was measured at 0.43 mm (p = 0.006) in the proximal fibula cohort, 0.19 mm (*p* = 0.21) in the middle fibula cohort, and 0.28 mm (*p* = 0.09) in the distal fibula cohort (Table 2). Differences in TFCS and MCS widening between cohorts was not statistically significant. On average, patients began weight-bearing 2.5 months postoperatively in the proximal fibula cohort, 2.5 months in the middle fibula cohort, and 2.7 months in the distal fibula cohort (Table 1).

**Table 2:** Radiographic syndesmosis measurements across follow-up

Radiographic measurement	Proximal fibula	Middle fibula	Distal fibula
Net change in MCS, average (SD) (mm)	0.43 ± 0.9	0.19 ± 0.8	0.28 ± 0.9
Net change in TFCS, average (SD) (mm)	0.50 ± 1.3	0.02 ± 1.3	0.67 ± 0.9

MCS = medial clear space, TFCS = tibiofibular clear space

## Discussion

In the management of Maisonneuve fractures, anatomic reduction of the distal tibiofibular joint with restoration of length and rotation of the fibula is a priority in treatment. Failure to restore fibular length results in significant lateral talar displacement, decreasing contact between the articular surfaces of the tibiotalar joint [11]. Shortening of the fibula is associated with painful ankle arthrosis and chronic instability, and quality of syndesmotic reduction is a significant predictor of functional outcomes [12, 13]. Successful treatment of Maisonneuve fractures thereby relies on adequate restoration of the ankle mortise.

In cases of proximal Maisonneuve injuries, direct reduction and fixation of the fibular fracture is not required for realignment of the syndesmosis. Restoration of the ankle mortise can often be achieved through fixation of the distal fibula to the tibia with trans-syndesmotic screws [6]. Several studies have demonstrated positive outcomes and maintenance of anatomic reduction in proximal Maisonneuve fractures treated with syndesmotic-only fixation [12, 14, 15]. Similarly, our cohort of proximal third Maisonneuve fractures demonstrated minimal MCS and TFCS widening across follow-up (Table 2). By demonstrating maintained anatomic reduction, these results strengthen the case for syndesmotic-only fixation of proximal Maisonneuve fractures.

While there is a consensus on treatment of proximal Maisonneuve fractures, no studies to date have evaluated outcomes in Maisonneuve fractures involving the middle third of the fibula. Current literature supports syndesmotic fixation in these injuries; however, the decision for open osteosynthesis of the fibula remains unclear [16]. Theoretical advantages of fibular plating in middle third fibula fractures include improved reduction and fixation strength. In a biomechanical study of 16 cadaveric legs, Ho et al. demonstrated increased rotational stability and load to failure following fibular plating when compared to syndesmotic-only fixation in mid-diaphyseal Maisonneuve fractures [9]. Though these mechanical advantages were noted, quality of reduction and functional outcomes were not assessed given the cadaveric nature of the study.

In our cohort, syndesmotic-only fixation of middle third Maisonneuve fractures resulted in restoration and maintenance of syndesmosis alignment across follow-up, as evidenced by TFCS and MCS measurements. Prior research has indicated that increases in syndesmosis measurements greater than 1.5 mm result in poor functional outcomes [17]. Changes in TFCS and MCS measurements in our middle third cohort were well below this threshold and not statistically significant. Additionally, TFCS and MCS changes were similar to those observed in the proximal and distal third fibula groups (Table 2). While plating the fibula may impart a biomechanical advantage, additional risk associated with larger dissection and exposure must also be considered. As demonstrated in our results, syndesmotic fixation alone is sufficient to restore syndesmosis alignment in these injuries.

In addition to maintained reduction, we report positive functional outcomes in middle third Maisonneuve fractures following syndesmotic-only fixation. There were no instances of infection, delayed wound healing, or loss of reduction. Reoperation for symptomatic hardware removal was the only observed complication and occurred in 8% (*n* = 1) of cases (Table 1). This reoperation rate was comparable to our proximal and distal third fibula cohorts and consistent with literature reported rates of reoperation for syndesmosis injuries following screw fixation [18]. Other outcome measures, including time to weight-bearing and ROM measurements, were also similar to averages reported in literature and did not vary significantly when compared to our proximal fibula cohort [12, 19]. Though the distal fibula cohort demonstrated greater plantar flexion ROM at final follow-up by five degrees, this difference is minute and likely of little clinical significance.

While we present positive outcomes following syndesmotic-only fixation of middle third Maisonneuve fractures, it is worth noting that a matched cohort study would be ideal in evaluating open fibular osteosynthesis with syndesmosis fixation versus syndesmosis fixation alone in these injuries. There are currently no studies to date presenting outcomes associated with open treatment of middle third Maisonneuve fractures, and there were no cases of open reduction with fibular plating in our dataset. In the absence of a fibular osteosynthesis cohort, we elected to compare outcomes and quality of reduction to proximal and distal third fibula fractures. Though not a matched cohort study, our findings of comparable reduction quality and complication rates across groups strengthen the case for syndesmotic-only fixation in middle third Maisonneuve fractures.

The limitations of this study include it being retrospective in nature, and it is therefore subject to limitations in data collection. All medical records were thoroughly reviewed to ensure accurate collection of data related to complications and outcomes. It is also worth noting that there are limitations to obtaining accurate TFCS and MCS measurements on radiographs and that changes recorded across follow-up may be impacted by variation in image quality or inconsistent patient positioning. Further, these images were largely non-weight-bearing radiographs, which limits the effects of weight-bearing on MCS and TFCS. However, patients did return to pain free weight-bearing at similar timepoints, thus reinforcing their equivalence. Finally, this study presents the largest sample size to date analyzing outcomes in middle third Maisonneuve fractures.

In conclusion, we present positive outcomes following syndesmotic-only fixation in the treatment of middle third Maisonneuve fractures. There were no documented cases of infection, loss of reduction, or wound healing complications, and rates of reoperation were comparable to our proximal and distal third fibula cohorts. In the management of Maisonneuve fractures, anatomic reduction of the distal tibiofibular joint with restoration of length and rotation of the fibula is a priority in treatment. All cohorts maintained adequate reduction of the ankle syndesmosis across follow-up, as evidenced by TFCS and MCS measurements. By demonstrating maintenance of anatomic reduction and similar rates of complications across cohorts, our results support the use of syndesmotic-only fixation in the treatment of middle third Maisonneuve fractures.
